# Anatomic Total Shoulder Arthroplasty Outcomes Were Not Negatively Affected by the COVID-19 Pandemic

**DOI:** 10.1055/s-0044-1785661

**Published:** 2024-06-22

**Authors:** Andrew J. Stevens, Akshar V. Patel, David Gibbs, Gregory Cvetanovich, Julie Y. Bishop, Ryan C. Rauck

**Affiliations:** 1 Departamento de Ortopedia, Ohio State University College of Medicine, Columbus, Ohio, Estados Unidos; 2 Departamento de Ortopedia, Jameson Crane Sports Medicine Institute, The Ohio State University Wexner Medical Center, Columbus, Ohio, Estados Unidos

**Keywords:** arthroplasty, replacement, COVID-19, postoperative period, shoulder

## Abstract

**Objective** To investigate whether patients undergoing anatomic total shoulder arthroplasty (ATSA) between January and March 2020 experienced different postoperative outcomes than patients in 2019. We hypothesized that patients in 2020 would have less access to physical therapy (PT) and experience different postoperative outcomes.

**Methods** Records from patients who received ATSA between January 1st, 2019, and March 17th, 2019, and January 1st, 2020, to March 17th, 2020, were analyzed. Patient data, including demographic information, range of motion (ROM), strength, and PT was collected and compared between the two groups. The 2020 patients were contacted by phone during October 2022 and patient-reported metrics were gathered.

**Results** The present study identified 24 patients in 2019 and 27 patients in 2020 who underwent ATSA during the specified time frame and had a minimum 1-year follow-up. Patients in 2019 experienced improvements in forward elevation (FE) ROM (125.4° to 146.7°; *p* = 0.008), external rotation (ER; 33.0° to 47.7°; *p* < 0.001), and internal rotation (IR; S1 to L4; *p* = 0.019). Patients in 2020 also experienced significant improvements in FE (120.2° to 141.1°; *p* = 0.009), ER (32.9° to 42.0°; *p* = 0.037), and IR (S1 to L3; *p* = 0.002). Patients in 2020 terminated PT earlier (2019: 125.8 days; 2020: 91.1 days; *p* = 0.046) and completed fewer sessions (2019: 21.4 sessions; 2020: 13.1 sessions; *p* = 0.003). At the final follow-up, patients in 2020 reported an average Visual Analogue Scale (VAS) pain score of 1.67 ± 1.1.

**Conclusion** Despite decreased PT, patients who underwent ATSA in 2020 had significant improvements in ROM and strength and were comparable to patients in 2019.

## Introduction

Anatomic total shoulder arthroplasty (ATSA) is the current gold-standard procedure for patients with end-stage glenohumeral arthritis who require shoulder replacement. 1 The volume of ATSA has been steadily increasing over the last few decades due to several factors, including the aging population and expanding indications for surgery. 2 Current rehabilitation protocols and timelines for ATSA vary between organizations; however, most follow similar principles of initial immobilization, reintroduction of passive range of motion (ROM), restoration of active ROM, and strength training. 1 Throughout the recovery process, physical therapy (PT) has been considered essential to achieve maximum shoulder function postoperatively. 3

The coronavirus disease 2019 (COVID-19) pandemic shutdowns interrupted the normal standard of care for patients receiving ATSA. The recent literature has illustrated that there was an associated decrease in surgical volume and hospital length of stay, with an increase in discharge-home rates and the use of telemedicine for follow-ups. 4 5 6 There is, however, a paucity of literature examining the effects of the pandemic shutdowns on long-term postoperative rehabilitation and patient outcomes. Understanding these effects could provide insight for future management, thus warranting further study.

The purpose of the present study is to examine the impact of the COVID-19 pandemic on patient PT attendance and surgical outcomes after ATSA at a tertiary medical center. We hypothesize that patients affected by the pandemic completed fewer PT sessions and likely experienced inferior ROM and strength testing at the final follow-up.

## Materials and Methods

### Patient Cohort

The present study was approved by the institutional review board (number: 2022H0150). Patients who had undergone ATSA between January 1st, 2019, and March 17th, 2019, and January 1st, 2020, to March 17th, 2020, were identified using the Current Procedural Terminology (CPT) codes 23470, 23472, 23473, and 23474, and screening operative reports for primary ATSA. All procedures were performed by four fellowship-trained orthopedic surgeons at a single tertiary care medical center. Patients undergoing revision ATSA and those with less than one year of postoperative follow-up were excluded from the analysis.

### Data Collection

Demographic, ROM, strength testing, and physical therapy data were collected from patients' medical records. Information on ROM included measurements of forward elevation (FE), external rotation (ER), and internal rotation (IR) from before and after surgery. Strength testing data was also collected from before and after surgery, and included measurements of FE, ER, and IR. Postoperative physical therapy information was collected, including the date of the first session, the date of the last session, and the number of completed sessions.

Subjective data was gathered by contacting all patients who had undergone surgery in 2020. Patients were contacted by phone during October 2022 and queried about their current Visual Analog Scale (VAS) pain score and Single Assessment Numeric Evaluation (SANE) score for each shoulder. Additionally, they were asked to report whether they felt the COVID-19 pandemic led to any unanticipated delays in postoperative PT and whether they thought it adversely affected their recovery. The SANE score was chosen as the primary patient-reported outcome metric (PROM) because of the literature supporting its interrater reliability with the American Shoulder and Elbow Surgeons (ASES) and Western Ontario Rotator Cuff (WORC) scores. 7

### Statistical Analysis

Demographic, preoperative, perioperative, and postoperative data from both the 2019 and 2020 cohorts were analyzed. Categorical data was analyzed using the Chi-squared test, along with the Fisher Exact test when appropriate. A two-sample *t* -test or Mann-Whitney U test was performed on continuous data depending on the normality of the sample. Normality was determined using the Kolmogorov-Smirnov test. Additionally, the F-test for equality of variance was used to compare variance between the two groups.

## Results

### Patient Demographic Data

A total of 51 patients with a minimum 1-year follow-up were included in the study. There were 24 patients in the 2019 cohort and 27 patients in the 2020 cohort. A total of six patients were excluded from analysis due to not meeting follow-up criteria. All of the patients underwent primary ATSA. There was no significant difference in follow-up length between the 2019 and 2020 cohorts (2019: mean – 2.2 years; standard deviation [SD] – ± 0.60 years; 2020: mean – 2.13 years; SD – ± 0.46 years; *p* = 0.56). Neither was there a significant difference in patient age at the time of surgery (2019: mean – 62.9 years; SD – ± 10.0 years; 2020: mean – 60.3 years; SD – ± 10.5; *p* = 0.39).

Of the 2019 cohort, 54.2% were male patients while 48.1% of the 2020 cohort were male subjects ( *p* = 0.68). The proportion of patients who received surgery on their right shoulder in 2019 was of 33.3% compared with 59.2% in 2020 ( *p* = 0.064). Finally, 16.7% of patients in the 2019 cohort had a history of surgery in the affected shoulder compared with 14.8% of patients in 2020 ( *p* = 0.86). Of the patients in 2019 who had received prior surgery, 1 underwent arthroscopic labral repair, 1 received arthroscopic debridement and decompression, and 2 underwent arthroscopic rotator cuff repair (RCR). From the 2020 cohort, prior surgeries included 2 arthroscopic labral repairs and 2 arthroscopic RCRs. A comparison of demographic data from each group can be found in Table 1 .

**Table 1:** Patient Demographic Comparison

Cohort	2019 ( *n* = 24)	2020 ( *n* = 27)	*p* -value
Age (years): mean ± SD	62.9 ± 10.0	60.3 ± 10.5	0.39
Follow-up (years): mean ± SD	2.2 ± 0.60	2.13 ± 0.46	0.56
Male patients (%)	54.2%	48.1%	0.68
Right shoulder (%)	33.3%	59.2%	0.064
History of surgery (%)	16.7%	14.8%	0.86

**Abbreviation:** SD, standard deviation.

**Note:** All significant *p* -values are in bold ( *p* < 0.05).

### Clinical Characteristics

Patients in the 2019 cohort experienced significant improvements in their ROM following ATSA. The FE was improved from 125.4° ± 31.9° to 146.7° ± 18.8° ( *p* = 0.008); ER measurements improved from 33.0° ± 15.6° to 47.7° ± 12.2° ( *p* < 0.001); and IR ability also improved from S1 to L4 ( *p* = 0.019). Patients in the 2019 cohort also experienced significant improvements in strength for FE (5- to 5; *p* = 0.031) and ER (5- to 5; *p* = 0.036), but not IR (5 to 5; *p* = 0.12). The average time from the date of surgery to the first postoperative appointment was of 16.1 (SD: ± 7.9) days.

Patients in the 2020 cohort also experienced significant improvements in their ROM: FE was improved from 120.2° ± 28.8° to 141.1° ± 25.9° ( *p* = 0.009); ER improved from 32.9° ± 16.5° to 42.0° ± 13.7° ( *p* = 0.037); and IR improved from S1 to L3 ( *p* = 0.002). Patients in the 2020 cohort did not experience significant improvements in strength for FE (5- to 5-; *p* = 0.38), ER (5- to 5-; *p* = 0.29), or IR (5 to 5; *p* = 0.76). The average time from the date of surgery to the first postoperative appointment was of 12.8 (SD: ± 1.6) days.

There were no significant differences in patient ROM or strength postoperatively between the two groups. A comparison of postoperative data can be found in Table 2 .

**Table 2:** Postoperative Clinical Outcomes Comparison

Cohort	2019 ( *n* = 24)	2020 ( *n* = 27)	*p* -value
Forward elevation (°): mean ± SD	146.7 ± 18.8	141.1 ± 25.9	0.39
External rotation (°): mean ± SD	47.7 ± 12.2	42. ± 13.7	0.13
Internal rotation	L4	L3	0.42
Strength – forward elevation	5/5	5-/5	0.17
Strength – external rotation	5/5	5-/5	0.18
Strength – internal rotation	5/5	5/5	0.12

**Abbreviation:** SD, standard deviation.

**Note:** All significant p-values are in bold ( *p* < 0.05).

Patients in the 2 groups did not differ significantly in the time taken to initiate PT (2019: 30.5 ± 12.9 days; 2020: 31.1 ± 12.2 days; *p* = 0.88). Patients in the 2020 cohort terminated PT more quickly (2019: 125.8 ± 70.7 days; 2020: 91.1 ± 47.0 days; *p* = 0.046) and completed fewer PT sessions compared with patients in the 2019 cohort (2019: 21.4 ± 10.8 sessions; 2020: 13.1 ± 8.4 sessions; *p* = 0.003). A comparison of PT data can be found in Table 3 .

**Table 3:** Physical Therapy Comparison

Cohort	2019 ( *n* = 24)	2020 ( *n* = 27)	*p* -value
Time from surgery to first session (days): mean ± SD	30.5 ± 12.9	31.1 ± 12.2	0.88
Time from first session to last session (days): mean ± SD	125.8 ± 70.7	91.1 ± 47.0	**0.046**
Number of sessions: mean ± SD	21.4 ± 10.8	13.1 ± 8.4	**0.003**

**Abbreviation:** SD, standard deviation.

**Note:** All significant *p* -values are in bold ( *p* < 0.05).

### Patient-Reported Outcomes and COVID-Related Delays

Of the 2020 cohort, 2 patients (7.4%) did not complete any PT after ATSA, 2 additional patients (7.4%) noted a delay in initiating PT, and 10 patients (37.0%) noted that their postoperative recovery was negatively affected by the COVID-19 pandemic. When patients were contacted by phone during October 2022, they reported an average VAS pain score of 1.67 ± 1.1, an average SANE score on the affected shoulder of 77.0 ± 15.8, and an average SANE score on the unaffected shoulder of 91.1 ± 10.4.

### Complications and Revisions

There was 1 complication in the 2019 cohort, and 1 complication in the 2020 cohort ( *p* = 0.95): the patient in the 2019 cohort ruptured his subscapularis and underwent revision surgery 3 months after the index procedure, and the patient from the 2020 cohort developed an infection and underwent revision surgery 11 months after their index ATSA.

## Discussion

The COVID-19 pandemic has changed clinical practice throughout numerous areas, yet the effects of this recent pandemic on patients undergoing ATSA remains unclear. Prior to this pandemic, normal postoperative management following ATSA included frequent PT with an emphasis on routine therapy as a means of restoring strength, function, and quality of life. 8 In contrast to reverse shoulder replacement, protecting the subscapularis repair is critical to obtain a good outcome after ATSA. In the current retrospective, single-center study, we analyzed and compared demographic, ROM, strength, and PT data between pre-pandemic and pandemic cohorts. The most important findings suggest that despite fewer PT sessions following ATSA, ROM, strength, and SANE scores were not significantly affected by the COVID-19 pandemic. These findings suggest that patients with limited access to PT care may similarly benefit from ATSA.

The pandemic demanded various adaptations, including an increase in telehealth visits and fewer PT sessions. 5 While events such as this recently-experienced COVID-19 pandemic are unpredictable, much can be learned and translated from the adaptations and clinical responses that ensued. Pertinent to the present study, large cohorts of patients who previously had ample access to non-essential medical care were abruptly quarantined and/or unable to continue with elements of their orthopedic treatments. While this disruption was unintended, the limitations experienced may in some regards reflect those of underserved populations and limited-resource nations where PT and high-quality postoperative care may not be routinely available. 9 As previously discussed, the objective of the present study was to investigate whether interruptions and delays in PT from the COVID-19 pandemic led to poorer outcomes following ATSA.

Parisien et al. 10 and Sabbagh et al. 5 were among the first authors to assess the utilization and impact of telehealth driven by the COVID-19 pandemic among orthopedic surgery patients. They observed significant telehealth utilization and no difference in patient satisfaction and PROMs compared with traditional in-person clinic follow-ups. Seetharam et al. 6 identified an increased number of outpatient TSAs at their institution during the pandemic, with a significant reduction in length of stay and 90-day readmission rate. Another study 4 looking at TSA covered by Medicare found an overall decrease in shoulder arthroplasty volume by 14% during the COVID-19 pandemic, with an average hospital stay decreasing by 16%, and no significant change in 30-day hospital readmission rates.

Secondary to mandated lockdowns and clinical judgment, numerous patients experienced limited access to their routine PT care. Wang et al. 9 reported similar findings, in which the COVID-19 shutdown decreased the availability of elective care. Similar to these findings, among our 2020 cohort, the COVID-19 pandemic led to earlier termination of PT and fewer overall PT sessions, culminating in 37% of patients citing the pandemic as a negative influence on their recovery following ATSA.

Historically, PT has been foundational in the perioperative restoration of strength and ROM. 11 12 Among our patients, strength and ROM favorably improved in both the 2019 and 2020 cohorts. Patients in 2019 and 2020 experienced comparable improvements in FE, ER and IR ROM. Regarding strength, patients in 2019 experienced significant improvements in FE and ER, but not IR. Notably, patients in 2020 did not experience significant improvements in FE, ER, or IR. These findings suggest that PT may be required to ensure adequate postoperative strength recovery, yet ROM may be restored similarly without PT. With decreased access to PT, our patients exhibited a similar ROM improvement and diminished strength recovery.

To further delineate these changes in strength and to continue quantifying the effects of the pandemic on ATSA, the SANE and VAS scores, as well as the PROMs, were obtained from the 2020 cohort. We found that 2 patients (7.4%) did not complete any PT, and 2 additional patients (7.4%) noted a delay in initiating their PT. A total of 10 patients (37.0%) noted that their postoperative recovery was negatively affected by the COVID-19 pandemic. When patients were contacted by phone during October 2022, they reported an average VAS pain score of 1.67, a SANE score on the affected shoulder of 77.0, and a SANE score on the unaffected shoulder of 91.1. The current literature estimates that the patient acceptable symptomatic state (PASS) for ATSA to be 75.5 for the SANE score and 1.5 for the VAS pain score. 12 13 Patients in our 2020 cohort approached or exceeded these benchmarks 2.5 years after surgery, suggesting that even patients with limited access to PT may still go on to achieve clinical significant improvements.

As there are many factors that influence patient recovery after surgery, it is difficult to say with certainty how much PT is required for patients to achieve satisfactory results. However, from our small cohort, we observed that patients who met criteria for acceptable improvement completed an average of 16.3 ± 7.2 PT sessions. This approximation was made by comparing our patients' improvement in FE ROM to the minimal clinically-important difference (MCID) of 23.1° ± 5.8° established by Simovitch et al. 14 and by calculating the number of sessions completed by those who exceeded the MCID threshold.

In summary, despite less PT and the perceived negative effect of the pandemic shutdowns, ATSA patients in 2020 demonstrated similar clinical and physical therapy outcomes when compared with a similar cohort of 2019 patients at our institution, aside from diminished strength recovery.

## Limitations

The present study has numerous limitations to address. Inherent to the design of this retrospective study, there is institutional selection and treatment bias that may be observed in the presented findings. Despite this limitation, best clinical practice recommendations were followed, and all orthopedic care was underseen by fellowship-trained surgeons from one academic medical center. Further, the present study is limited in its sample size and selection bias, which further limits the statistical power and generalizability of the presented observations. Finally, PT protocols may vary greatly across institutions, and this may influence the lack of observed difference between cohorts.

## Conclusion

The COVID-19 pandemic shutdowns led to many changes in postoperative management of ATSA patients. As a whole, patients in the 2020 cohort ended PT more quickly and completely fewer sessions, with 37% stating that their recovery was adversely affected by the pandemic. Despite these setbacks, patients in the cohort still obtained satisfactory outcomes in ROM, SANE, and VAS pain scores. These findings suggest that patients who have limited access to PT may still go on to achieve acceptable results following ATSA.
