# Supplementary material for: Anatomic Total Shoulder Arthroplasty Outcomes Were Not Negatively Affected by the COVID-19 Pandemic
Source: Rev Bras Ortop (Sao Paulo). 2024 Jun 22;59(3):e429–34. doi: 10.1055/s-0044-1785661 (PMC11193572; doi:10.1055/s-0044-1785661)
Supplement: Related texts

# Research article: Os desfechos da artroplastia anatômica total do ombro não foram afetados negativamente pela pandemia de Covid-19

**Keywords:** artroplastia de substituição, Covid-19, ombro, período pós-operatório

## Abstract

**Objetivo** Investigar se os pacientes submetidos à artroplastia total anatômica do ombro (ATAO) entre janeiro e março de 2020 tiveram resultados pós-operatórios diferentes dos pacientes operados em 2019. Nossa hipótese é a de que os pacientes de 2020 teriam menos acesso à fisioterapia (FT) e apresentariam desfechos pós-operatórios diferentes.

**Métodos** Foram analisados os prontuários de pacientes submetidos à ATAO de 1° de janeiro de 2019 a 17 de março de 2019 e de 1° de janeiro de 2020 a 17 de março de 2020. Os dados dos pacientes, incluindo informações demográficas, amplitude de movimento (ADM), força e FT foram coletados e comparados entre os dois grupos. Os pacientes de 2020 foram contatados por telefone durante outubro de 2022, e as métricas relatadas por eles pacientes foram coletadas.

**Resultados** Este estudo identificou 24 pacientes em 2019 e 27 pacientes em 2020 que foram submetidos à ATAO durante o período especificado e tiveram acompanhamento mínimo de 1 ano. Os pacientes em 2019 apresentaram melhoras na ADM de elevação anterior (EA; 125,4° a 146,7°; *p* = 0,008), rotação externa (RE; 33,0° a 47,7°; *p* < 0,001) e rotação interna (RI; S1 a L4; *p* = 0,019). Os pacientes em 2020 também apresentaram melhoras significativas de EA (120,2° a 141,1°; *p* = 0,009), RE (32,9° a 42,0°; *p* = 0,037) e RI (S1 a L3; *p* = 0,002). Os pacientes de 2020 terminaram a FT mais cedo (2019: 125,8 dias; 2020: 91,1 dias; *p* = 0,046) e fizeram menos sessões (2019: 21,4 sessões; 2020: 13,1 sessões; *p* = 0,003). No acompanhamento final, os pacientes de 2020 relataram uma pontuação média de dor na Escala Visual Analógica (EVA) de 1,67 ± 1,1.

**Conclusão** Apesar da menor realização de FT, os pacientes submetidos à ATAO em 2020 apresentaram melhoras significativas na ADM e na força e foram comparáveis aos pacientes de 2019.

## Introdução

A artroplastia total anatômica do ombro (ATAO) é o procedimento padrão-ouro atual para pacientes com artrite glenoumeral em estágio terminal que necessitam de substituição do ombro. 1 O volume da ATAO tem aumentado constantemente nas últimas décadas devido a vários fatores, incluindo o envelhecimento da população e indicações crescentes para cirurgia. 2 Os protocolos de reabilitação atuais e os cronogramas para ATAO variam entre as organizações; no entanto, a maioria segue princípios semelhantes de imobilização inicial, reintrodução da amplitude de movimento (ADM) passiva, restauração da ADM ativa e treinamento de força. 1 Durante todo o processo de recuperação, a fisioterapia (FT) tem sido considerada essencial para alcançar a função máxima do ombro no período pós-operatório. 3

As paralisações devido à pandemia de doença do coronavírus 2019 ( *coranavirus disease 2019* , Covid-19, em inglês) interromperam o padrão normal de atendimento aos pacientes submetidos à ATAO. A literatura recente mostrou uma diminuição no volume cirúrgico e no tempo de internação hospitalar, e um aumento nas taxas de alta hospitalar e no uso da telemedicina para acompanhamento. 4 5 6 Há, no entanto, uma escassez de estudos na literatura sobre os efeitos das paralisações pandêmicas na reabilitação pós-operatória de longo prazo e nos desfechos dos pacientes. A compreensão desses efeitos poderia fornecer informações para o manejo futuro, o que justificaria, portanto, um estudo mais aprofundado.

O objetivo deste estudo foi examinar o impacto da pandemia de Covid-19 no atendimento a pacientes fisioterápicos e nos desfechos cirúrgicos após a ATAO em um centro médico terciário. Nossa hipótese é a de que os pacientes afetados pela pandemia realizaram menos sessões de FT e provavelmente apresentaram ADM e testes de força inferiores no último acompanhamento.

## Materiais e Métodos

## Coorte de Pacientes

Este estudo foi aprovado pelo Comitê de Ética institucional (número: 2022H0150). Pacientes submetidos à ATAO de 1 ^o^ de janeiro de 2019 a 17 de março de 2019 e de 1 ^o^ de janeiro de 2020 a 17 de março de 2020 foram identificados usando os códigos 23470, 23472, 23473 e 23474 da Terminologia Processual Atual (CPT, do inglês *Current Procedural Terminology* ) e relatórios cirúrgicos de triagem para ATAO primária. Todos os procedimentos foram realizados em um único centro médico terciário por quatro cirurgiões ortopédicos treinados. Pacientes submetidos à ATAO de revisão e pacientes com menos de um ano de acompanhamento pós-operatório foram excluídos da análise.

## Coleta de Dados

Dados demográficos, de ADM, de testes de força e de FT foram coletados dos prontuários médicos dos pacientes. As informações sobre ADM= incluíram medidas de elevação anterior (EA), rotação externa (RE) e rotação interna (RI) antes e depois da cirurgia. Dados de testes de força também foram coletados antes e depois da cirurgia, e incluíram medidas de EA, RE e RI. Informações sobre FT pós-operatória foram coletadas, incluindo data da primeira sessão, data da última sessão e número de sessões concluídas.

Os dados subjetivos foram coletados por meio de contato com todos os pacientes submetidos à cirurgia em 2020. Os pacientes foram contatados por telefone durante outubro de 2022 e questionados sobre sua pontuação atual de dor segundo Escala Visual Analógica (EVA) e a pontuação de *Single Assessment Numeric Evaluation* (SANE, em inglês) de cada ombro. Além disso, pedimos que relatassem se sentiam que a pandemia de Covid-19 havia acarretado quaisquer atrasos imprevistos na FT pós-operatória, e se achavam que isso afetava negativamente a sua recuperação. A pontuação SANE foi escolhida como a principal métrica de desfecho relatado pelo paciente (MDRP) porque a literatura dá apoio à sua confiabilidade interobservadores com as pontuações *American Shoulder and Elbow Surgeons* (ASES, em inglês) e *Western Ontario Rotator Cuff* (WORC, em inglês). 7

## Análise Estatística

Foram analisados dados demográficos, pré-operatórios, perioperatórios e pós-operatórios das coortes de 2019 e 2020. Os dados categóricos foram analisados por meio dos testes de Qui-quadrado, além do teste exato de Fisher quando apropriado. Dados contínuos foram submetidos ao teste *t* para duas amostras ou ao teste U de Mann-Whitney, dependendo da normalidade da amostra, determinada pelo teste de Kolmogorov-Smirnov. Além disso, o teste F de igualdade de variância foi utilizado para a comparação da variância entre os dois grupos.

## Resultados

## Dados Demográficos dos Pacientes

No total, 51 pacientes com acompanhamento mínimo de 1 ano foram incluídos no estudo. Havia 24 pacientes na coorte de 2019 e 27 pacientes na coorte de 2020. Ao todo, 6 pacientes foram excluídos da análise por não atenderem aos critérios de acompanhamento. Todos os pacientes foram submetidos à ATAO primária. Não houve diferença significativa no tempo de acompanhamento entre a coorte de 2019 e 2020 (2019: média – 2,2 anos; desvio-padrão [DP] – ± 0,60 ano; 2020: média – 2,13 anos; DP – ± 0,46 ano; *p* = 0,56). Tampouco houve diferença significativa na idade dos pacientes no momento da cirurgia (2019: média – 62,9 anos; DP – ± 10,0 anos; 2020: média – 60,3 anos; DP – ± 10,5 anos; *p* = 0,39).

Na coorte de 2019, 54,2% dos pacientes eram do sexo masculino, ao passo que 48,1% dos indivíduos da coorte de 2020 eram do sexo masculino ( *p* = 0,68). A proporção de pacientes operados no ombro direito em 2019 foi de 33,3% em comparação a 59,2% em 2020 ( *p* = 0,064). Por fim, 16,7% dos pacientes da coorte de 2019 tinham histórico de cirurgia no ombro acometido, em comparação a 14,8% dos pacientes de 2020 ( *p* = 0,86). Dos pacientes de 2019 com histórico de cirurgia, 1 foi submetido a reparo labral artroscópico, 1 recebeu desbridamento e descompressão artroscópicos e 2 foram submetidos a reparo artroscópico do manguito rotador (RMR). Da coorte de 2020, as cirurgias anteriores incluíram 2 reparos labrais artroscópicos e 2 RMRs artroscópicos. A Tabela 1 compara os dados demográficos de cada grupo.

**Tabela 1:** Comparação dos dados demográficos de pacientes

Coorte	2019 ( *n* = 24)	2020 ( *n* = 27)	Valor de *p*
Idade (anos): média ± DP	62,9 ± 10,0	60,3 ± 10,5	0,39
Acompanhamento (anos) : média ± DP	2,2 ± 0,60	2,13 ± 0,46	0,56
Homens (%)	54,2%	48,1%	0,68
Ombro direito (%)	33,3%	59,2%	0,064
Histórico de cirurgia (%)	16,7%	14,8%	0,86

**Abreviatura:** DP, desvio padrão.

**Nota:** Todos os valores de *p* significativos estão em negrito ( *p* < 0,05).

## Características Clínicas

Os pacientes da coorte de 2019 apresentaram melhoras significativas na ADM após a ATAO: a EA melhorou de 125,4° ± 31,9° para 146,7° ± 18,8° ( *p* = 0,008); a RE melhorou de 33,0° ± 15,6° para 47,7° ± 12,2° ( *p* < 0,001); e a RI também melhorou, de S1 para L4 ( *p* = 0,019). Os pacientes da coorte de 2019 também apresentaram melhoras significativas na força de EA (5- a 5; *p* = 0,031) e RE (5- a 5; *p* = 0,036), mas não de RI (5 a 5; *p* = 0,12). O tempo médio desde a data da cirurgia até a primeira consulta pós-operatória foi de 16,1 (DP: ± 7,9) dias.

Os pacientes da coorte de 2020 também apresentaram melhoras significativas na ADM após a ATAO: a EA melhorou de 120,2° ± 28,8° para 141,1 ± 25,9° ( *p* = 0,009); a RE melhorou de 32,9° ± 16,5° para 42,0° ± 13,7° ( *p* = 0,037); e a RI melhorou de S1 para L3 ( *p* = 0,002). Os pacientes da coorte de 2020 não apresentaram melhoras significativas na força de EA (5- a 5-; *p* = 0,38), RE (5- a 5-; *p* = 0,29) ou RI (5 a 5; *p* = 0,76). O tempo médio desde a data da cirurgia até a primeira consulta pós-operatória foi de 12,8 (DP: ± 1,6) dias.

Não houve diferenças significativas na ADM ou força dos pacientes dos dois grupos no período pós-operatório. A Tabela 2 compara os dados pós-operatórios.

**Tabela 2:** Comparação dos desfechos clínicos pós-operatórios

Coorte	2019 ( *n* = 24)	2020 ( *n* = 27)	Valor de *p*
Elevação anterior (°): média ± DP	146,7 ± 18,8	141,1 ± 25,9	0,39
Rotação externa (°): média ± DP	47,7 ± 12,2	42,0 ± 13,7	0,13
Rotação interna	L4	L3	0,42
Força – elevação anterior	5/5	5-/5	0,17
Força – rotação externa	5/5	5-/5	0,18
Força – rotação interna	5/5	5/5	0,12

**Abreviatura:** DP, desvio padrão.

**Nota:** Todos os valores de *p* significativos estão em negrito ( *p* < 0,05).

Os pacientes dos 2 grupos não diferiram significativamente no tempo de início da FT (2019: 30,5 ± 12,9 dias; 2020: 31,1 ± 12,2 dias; *p* = 0,88). Os pacientes da coorte de 2020 encerraram a FT mais rapidamente (2019: 125,8 ± 70,7 dias; 2020: 91,1 ± 47,0 dias; *p* = 0,046) e realizaram menos sessões de FT em comparação aos pacientes da coorte de 2019 (2019: 21,4 ± 10,8 sessões; 2020: 13,1 ± 8,4 sessões; *p* = 0,003). A Tabela 3 compara os dados de FT.

**Tabela 3:** Comparação da fisioterapia

Coorte	2019 ( *n* = 24)	2020 ( *n* = 27)	Valor de *p*
Tempo entre a cirurgia e a primeira sessão (dias): média ± DP	30,5 ± 12,9	31,1 ± 12,2	0,88
Tempo entre a primeira e a última sessão (dias): média ± DP	125,8 ± 70,7	91,1 ± 47,0	**0,046**
Número de sessões: média ± DP	21,4 ± 10,8	13,1 ± 8,4	**0,003**

**Abreviatura:** DP, desvio padrão.

**Nota:** Todos os valores de *p* significativos estão em negrito ( *p* < 0,05).

## Desfechos Relatados pelo Paciente e Atrasos Relacionados à Covid

Da coorte de 2020, 2 pacientes (7,4%) não realizaram nenhuma FT após a ATAO. Outros 2 pacientes (7,4%) notaram atraso no início da FT, e 10 pacientes (37,0%) notaram que sua recuperação pós-operatória foi afetada negativamente pela pandemia de Covid-19. Quando os pacientes foram contatados por telefone em outubro de 2022, relataram uma pontuação média de dor na EVA de 1,67 ± 1,1, uma pontuação média SANE no ombro acometido de 77,0 ± 15,8 e uma pontuação média SANE no ombro não acometido de 91,1 ± 10,4.

## Complicações e Revisões

Houve 1 complicação na coorte de 2019 e 1 complicação na coorte de 2020 ( *p* = 0,95). O paciente da coorte de 2019 rompeu o subescapular e foi submetido à cirurgia de revisão 3 meses após o procedimento índice. O paciente da coorte de 2020 desenvolveu uma infecção e foi submetido a uma cirurgia de revisão 11 meses após a ATAO índice.

## Discussão

A pandemia de Covid-19 mudou a prática clínica em diversas áreas, mas seus efeitos em pacientes submetidos à ATAO ainda são obscuros. Antes desta pandemia, o manejo pós-operatório normal após ATAO incluía FT frequente com ênfase na terapia de rotina para restauro de força, função e qualidade de vida. 8 Diferentemente da artroplastia reversa do ombro, a proteção do reparo do subescapular é fundamental para a obtenção de um bom resultado após a ATAO. Neste estudo retrospectivo e unicêntrico, analisamos e comparamos dados demográficos, ADM, força e FT entre coortes pré-pandêmicas e pandêmicas. As descobertas mais importantes sugerem que, apesar do número menor de sessões de FT após a ATAO, a ADM, a força e as pontuações SANE não foram significativamente afetadas pela pandemia de Covid-19. Estes resultados sugerem que os pacientes com acesso limitado à FT podem igualmente se beneficiar da ATAO.

A pandemia exigiu várias adaptações, incluindo um aumento nas consultas de telessaúde e menos sessões de FT. 5 Embora acontecimentos como esta recente pandemia de Covid-19 sejam imprevisíveis, muito pode ser aprendido e traduzido a partir das adaptações e respostas clínicas que se seguiram. Pertinente a este estudo, grandes coortes de pacientes que antes tinham amplo acesso a cuidados médicos não essenciais foram abruptamente colocados em quarentena e/ou incapazes de continuar com elementos dos seus tratamentos ortopédicos. Embora esta perturbação não tenha sido intencional, as limitações experimentadas podem, em alguns aspectos, refletir as de populações mal servidas e de nações com recursos limitados, em que a FT e os cuidados pós-operatórios de alta qualidade podem não estar disponíveis rotineiramente. 9 Como já discutido, o objetivo deste estudo foi o de investigar se as interrupções e atrasos na FT devido à pandemia de Covid-19 levaram a desfechos piores após a ATAO.

Parisien et al. 10 e Sabbagh et al. 5 estiveram entre os primeiros autores a avaliar a utilização e o impacto da telessaúde impulsionada pela pandemia de Covid-19 entre pacientes de cirurgia ortopédica. Eles observaram uma utilização significativa da telessaúde e nenhuma diferença na satisfação do paciente e nas MDRPs em comparação ao acompanhamento clínico presencial tradicional. Seetharam et al. 6 identificaram um aumento no número de ATAO ambulatoriais em sua instituição durante a pandemia, com redução significativa do tempo de internação e da taxa de readmissão em 90 dias. Outro estudo 4 que analisou a ATAO coberta pelo Medicare observou uma diminuição geral no volume de artroplastia de ombro em 14% durante a pandemia de Covid-19, com uma redução média de internação hospitalar de 16% e nenhuma mudança significativa nas taxas de readmissão hospitalar em 30 dias.

Secundariamente aos bloqueios obrigatórios e ao julgamento clínico, numerosos pacientes tiveram acesso limitado à FT de rotina. Wang et al. 9 relataram achados semelhantes, em que o confinamento devido à Covid-19 diminuiu a disponibilidade de cuidados eletivos. Consistente com esses achados, em nossa coorte de 2020, a pandemia de Covid-19 levou ao término mais precoce da FT e à redução do número de sessões de FT, o que levou 37% dos pacientes a citar a pandemia como uma influência negativa em sua recuperação após a ATAO.

Historicamente, a FT tem sido fundamental na restauração perioperatória da força e da ADM. 11 12 Em nossa coorte, a força e a ADM melhoraram favoravelmente nas coortes de 2019 e 2020. Os pacientes de 2019 e 2020 apresentaram melhoras comparáveis nas ADMs de EA, RE e RI. Em relação à força, os pacientes de 2019 apresentaram melhoras significativas de EA e RE, mas não de RI. Notavelmente, os pacientes de 2020 não apresentaram melhoras significativas de EA, RE ou RI. Esses achados sugerem que a FT pode ser necessária para garantir a recuperação adequada da força pós-operatória, mas a ADM pode ser restaurada de forma semelhante sem a FT. Com a diminuição do acesso à FT, nossos pacientes exibiram melhora semelhante na ADM e menor recuperação de força.

Para delinear melhor essas mudanças na força e continuar a quantificar os efeitos da pandemia na ATAO, as MDRPs e as pontuações SANE e EVA foram obtidas da coorte de 2020. Descobrimos que 2 pacientes (7,4%) não fizeram nenhuma FT, e outros 2 (7,4%) notaram atraso no início da FT. Ao todo, 10 pacientes (37,0%) observaram que sua recuperação pós-operatória foi afetada negativamente pela pandemia de Covid-19. Quando os pacientes foram contatados por telefone em outubro de 2022, relataram uma pontuação média de dor na EVA de 1,67, uma pontuação SANE no ombro acometido de 77,0, e uma pontuação SANE no ombro não acometido de 91,1. A literatura atual estima que o estado sintomático aceitável do paciente (PASS, do inglês *patients' acceptable symptom state* ) para ATAO seja de 75,5 para o escore SANE e de 1,5 para a EVA de dor. 12 13 Os pacientes de nossa coorte de 2020 apresentaram valores próximos ou acima destes valores de referência 2,5 anos após a cirurgia, o que sugere que mesmo pacientes com acesso limitado à FT ainda podem alcançar melhoras clínicas significativas.

Como existem muitos fatores que influenciam a recuperação do paciente após a cirurgia, é difícil dizer com certeza quanto de FT é necessário para a obtenção de desfechos satisfatórios. No entanto, em nossa pequena coorte, observamos que os pacientes que preencheram os critérios para melhora aceitável fizeram, em média, 16,3 ± 7,2 sessões de FT. Essa aproximação foi feita comparando a melhora da ADM de EA de nossos pacientes com a diferença mínima clinicamente importante (MCDI, do inglês *minimal clinically important difference* ) de 23,1° ± 5,8° estabelecida por Simovitch et al. 14 e calculando o número de sessões concluídas por aqueles que excederam o limite da MCID.

Em suma, apesar de menos FT e do efeito negativo percebido por conta das paralisações pandêmicas, os pacientes submetidos à ATAO em 2020 demonstraram desfechos clínicos e fisioterapêuticos semelhantes quando comparados a uma coorte similar de pacientes de 2019 em nossa instituição, mas com menor recuperação de força.

## Limitações

Este estudo tem inúmeras limitações a serem abordadas. Inerente ao delineamento retrospectivo, vieses de seleção institucional e tratamento podem ser observados. Apesar desta limitação, as melhores recomendações de práticas clínicas foram seguidas, e todos os cuidados ortopédicos foram supervisionados por cirurgiões treinados em um centro médico acadêmico. Além disso, o estudo apresentado é limitado no tamanho da amostra e no viés de seleção, o que diminui ainda mais o poder estatístico e a generalização das observações apresentadas. Por fim, os protocolos de FT podem variar muito entre as instituições, o que pode influenciar a ausência de diferença observada entre as coortes.

## Conclusão

As paralisações decorrentes da pandemia de COVID-19 levaram a muitas mudanças no manejo pós-operatório de pacientes submetidos à ATAO. De modo geral, os pacientes da coorte de 2020 terminaram a FT mais rapidamente e com menos sessões; 37% destes indivíduos afirmaram que a sua recuperação foi afetada negativamente pela pandemia. Apesar desses contratempos, os pacientes da coorte ainda obtiveram resultados satisfatórios em termos de ADM e das pontuações SANE e de dor na EVA. Esses achados sugerem que os pacientes com acesso limitado à FT ainda podem alcançar desfechos aceitáveis após a ATAO. 1
